# A homeobox protein Phx1 regulates long-term survival and meiotic sporulation in *Schizosaccharomyces pombe*

**DOI:** 10.1186/1471-2180-12-86

**Published:** 2012-05-30

**Authors:** Ji-Yoon Kim, Eun-Soo Kwon, Jung-Hye Roe

**Affiliations:** 1 Laboratory of Molecular Microbiology, School of Biological Sciences, and Institute of Microbiology, Seoul National University, Seoul, 151-742, South Korea; 2 Program in Gene Function and Expression, University of Massachusetts Medical School, Worcester, MA, 01605, USA

## Abstract

**Background:**

In the fission yeast *Schizosaccharomyces pombe*, the *phx1*^+^ (pombe homeobox) gene was initially isolated as a multi-copy suppressor of lysine auxotrophy caused by depletion of copper/zinc-containing superoxide dismutase (CuZn-SOD). Overproduction of Phx1 increased the synthesis of homocitrate synthase, the first enzyme in lysine biosynthetic pathway, which is labile to oxidative stress. Phx1 has a well conserved DNA-binding domain called homeodomain at the N-terminal region and is predicted to be a transcription factor in *S. pombe*. However, its role has not been revealed in further detail. Here we examined its expression pattern and the phenotype of its null mutant to get clues on its function.

**Results:**

Fluorescence from the Phx1-GFP expressed from a chromosomal fusion gene demonstrated that it is localized primarily in the nucleus, and is distinctly visible during the stationary phase. When we replaced the N-terminal homeobox domain of Phx1 with the DNA binding domain of Pap1, a well-characterized transcription factor, the chimeric protein caused the elevation of transcripts from Pap1-dependent genes such as *ctt1*^*+*^ and *trr1*^*+*^, suggesting that Phx1 possesses transcriptional activating activity when bound to DNA. The amount of *phx1*^*+*^ transcripts sharply increased as cells entered the stationary phase and was maintained at high level throughout the stationary phase. Nutrient shift down to low nitrogen or carbon sources caused *phx1*^*+*^ induction during the exponential phase, suggesting that cells need Phx1 for maintenance function during nutrient starvation. The *Δphx1* null mutant showed decreased viability in long-term culture, whereas overproduction of Phx1 increased viability. Decrease in long-term survival was also observed for *Δphx1* under N- or C-starved conditions. In addition, *Δphx1* mutant was more sensitive to various oxidants and heat shock. When we examined sporulation of the *Δphx1/Δphx1* diploid strain, significant decrease in the formation of meiotic spores was observed.

**Conclusions:**

Phx1 is a transcriptional regulator whose synthesis is elevated during stationary phase and by nutrient starvation in *S. pombe*. It supports long-term survival and stress tolerance against oxidation and heat, and plays a key role in the formation of meiotic spores.

## Background

Homeobox genes, first identified to control development in *Drosophila* species, encode highly conserved domains of about 60 amino acids, which comprise helix-turn-helix DNA-binding motif [1]. Homeobox genes are found in various organisms from yeast to vertebrates, and most homeodomain-containing proteins are believed to act as transcriptional factors [2]. In vertebrates, Hox proteins participate in various differentiation programs such as limb development [3] and also in regulating cell cycle, apoptosis and cancer [4,5]. In fungi, homeobox genes are best known to determine mating-types in *Saccharomyces cerevisiae*[6]*, Schizosaccharomyces pombe*[7], as well as in other fungi [8]. Control of phosphate starvation response, hyphal formation, or cell cycle by homeobox genes has also been reported [9-11].

In *S. pom*, there are three homeobox family genes; the mating type control gene *matPi*[7], *yox1*^*+*^ whose product is a regulator of G1/S transition of the cell cycle [11,12], and *phx1*^*+*^ that was initially isolated as a high-copy suppressor of the growth defect caused by mutation in Cu, Zn-containing superoxide dismutase (CuZnSOD) production [13]. Depletion of CuZnSOD caused lysine auxotrophy, and the overproduction of Phx1 increased the synthesis of homocitrate synthase, the first enzyme in lysine biosynthetic pathway. Since homocitrate synthase is labile to oxidative stress, it has been postulated that Phx1 may serve as a transcriptional regulator that increases the fitness of *S. pombe* cells against oxidative stress [13]. However, no further information about the role of Phx1 has been available. In this study, we examined the expression pattern of the *phx1* gene, and its mutant phenotype to investigate its function. We found that Phx1 plays an important role during the stationary phase when nutrients are low, enabling long-term survival, stress tolerance, and meiotic sporulation. Supporting evidence for its action as a transcriptional regulator has also been presented.

## Results and discussion

### Phx1 is a homeodomain protein localized primarily in the nucleus

**Figure 1:**
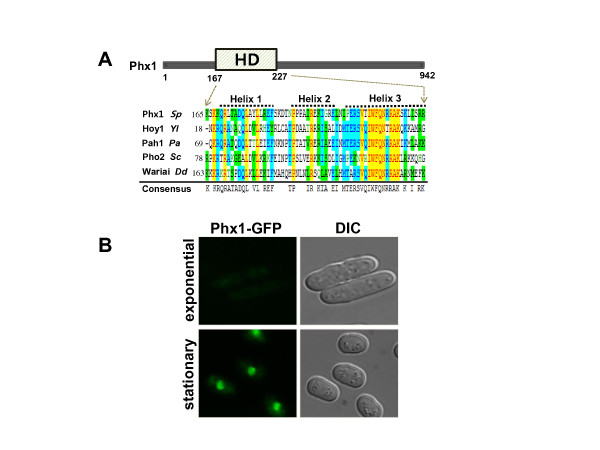
**Sequence composition of the conserved homeodomain in Phx1 and its subcellular localization.**(**A**) Multiple sequence alignment of the homeodomain (HD; 167–227) of Phx1 with those of other fungi; Hoy1p of *Yarrowia lipolytica* (*Yl*), Pah1p of *Podospora anserina* (*Pa*), Pho2p of *S. cerevisiae* (*Sc*), Wariai of *Dictyostelium discoideum* (*Dd*). The sequences were aligned using Vector NTI AlignX program (Invitrogen Co.). The three α-helices are indicated above and the consensus was shown at the bottom. The sequences were retrieved from the GenBank database. [CAA93700, CAA84415, CAC16792, CAA64906, AAB92245 for Phx1, Hoy1p, Pah1p, Pho2p, Wariai respectively]. (**B**) Localization of Phx1-GFP. Cells containing the chromosomally integrated fusion gene for Phx1-GFP were grown in liquid EMM at 30°C. Aliquots taken during the exponential (OD_600_ of 1, at around 18 h culture) and stationary (OD_600_ of 8–9, at around 42 h culture) phases were examined for fluorescence and DIC images by fluorescence microscopy (Axiovert 200 M, Carl Zeiss).

Phx1 is a large protein of 942 amino acids (103.9 kDa), with conserved homeodomain (a.a. 167–227). The homeodomain consists of a flexible stretch of several residues (N-terminal arm) followed by three α-helices [14]. Multiple alignment of Phx1 and other related proteins from *Yarroia lipolytica* (Hoy1p), *Podospora anserina* (Pah1p), *S. cerevisiae* (Pho2p), and *Dictyostelium discoideum* (Wariai), indicates that the homeodomains are highly conserved, especially in the third helix (Figure 1A). Many eukaryotic homeodomain proteins with similar DNA-binding motifs can bind the same DNA sequences *in vitro*. However, these proteins function in different stages and regions, implying that their regulatory specificity can be determined through the combinational interaction with other transcriptional regulators. Besides the homeodomain region, a small stretch of residues (from a.a. 520 to 566) was found to be conserved, sharing about 40% identical residues with the corresponding region of Pho2. Interestingly, this region was reported to be involved in interaction with binding partners of Pho2P such as Pho4p, Bas1p, and Swi5p in *S. cerevisiae*[15,16]*.* It implies that Phx1 may have binding partners and related regulatory mechanisms as revealed in the action of transcription factor Pho2p in *S. cerevisiae*.

In order to examine its expression and subcellular localization, we made a construct to encode Phx1 with C-terminally fused GFP, by integrating the fused gene into the chromosome. Cells were grown in Edinburgh minimal medium (EMM) and examined for fluorescence at different growth phases. The GFP fluorescence began visible at late exponential phase and became very evident in the nucleus during the stationary phase (Figure 1B). The nuclear localization of Phx1 is in agreement with the genome-scale analysis data of protein localization in *S. pombe*[17].

### Phx1 contains the ability for transcriptional activation

**Figure 2:**
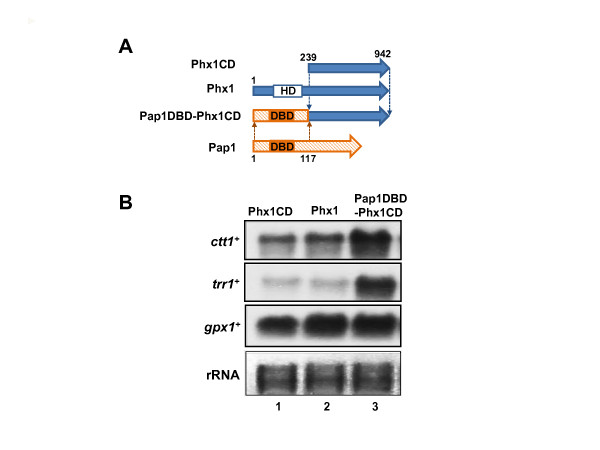
**Transcriptional activation by DNA-bound Phx1.** (**A**) Construction of Pap1-Phx1 chimeric protein where the N-terminal homeodomain region of Phx1 was replaced with the DNA-binding domain (DBD) of Pap1. The domain structure of full-length Phx1, N-terminally deleted one (Phx1CD; 239–942 aa), and the chimeric form (Pap1DBD-Phx1CD) that contains N-terminal region (1–117) of Pap1. (**B**) Freshly grown wild type (ED665) cells harboring pREP42-*phx1CD* (lane 1), pREP42-*phx1*^*+*^ (lane 2), or pREP42-*pap1DBD-phx1CD* (lane 3) were inoculated in liquid EMM media, and grown to OD_600_ of 1.0. Following cells harvest, RNA samples were analyzed by Northern blot, using gene-specific probes for *ctt1*^*+*^, *trr1*,^*+*^ or *gpx1*^*+*^ transcripts that encode catalase, thioredoxin reductase, or glutathione peroxidase, respectively. The ribosomal RNAs for each sample were visualized for loading control.

Many homeodomain-containing proteins are able to bind to DNA and act as a transcription factor. In order to investigate the DNA binding ability of Phx1 protein, we purified the N- terminal polypeptide fragment containing homeodomain (Phx1-ND; a.a. 1–431) as a fusion form with GST (glutathione-S-transferase) from *E. coli* and examined whether it can bind to *S. pombe* genomic DNA fragment. When Phx1-ND-GST was bound to glutathione Sepharose 4B column, *S. pombe* DNA was retained in the column whereas nearly no retention was observed in the absence of protein, suggesting that Phx1 is a DNA-binding protein (data not shown). However, the specificity of the bound DNA was not readily extractable. In the absence of information on its specific target sequence, we moved on to find whether it has the ability to activate transcription when bound to a promoter region. For this purpose, we created a recombinant, where the N-terminal homeodomain region (from a.a. 1–238) of Phx1 was swapped with the N-terminal DNA(a space) binding domain (a.a. 1–117) of Pap1, a well-studied transcription factor with known target genes [18] (Figure 2A). The chimeric protein was expressed from a multi-copy plasmid pREP42 in *S. pombe* cells, and the level of Pap1-dependent *ctt1*^*+*^ and *trr1*^*+*^ transcripts as well as Pap1-independent *gpx1*^*+*^ gene was examined by Northern analysis (Figure 2B). As a control, RNA samples from cells that express either the full-length (lane 2) or C-terminal domain of Phx1 (Phx1CD; a.a. 239–942; lane 1) were analyzed in parallel. The results in Figure 2B demonstrate that the chimeric construct that can bind to Pap1-binding sites elevated transcripts of Pap1 target genes (*ctt1*^*+*^ and *trr1*^*+*^) without affecting transcripts from Pap1-independent *gpx1*^*+*^ gene. We separately confirmed that overproduction of Pap1 in this strain increased the expression of *trr1*^*+*^ and *ctt1*^*+*^ genes by about 1.7- and 3.2-fold, respectively, whereas that of *gpx1*^*+*^ was not significantly changed (0.9-fold), when monitored by quantitative real-time PCR. These results indicate that the C-terminal two-thirds of Phx1 (a.a. 239–942) most likely contain a region that activates transcription when tethered nearby to the promoter. This supports the proposal that Phx1 is likely to be a transcription factor. Whether Phx1 can act alone or needs interaction with other regulators remains to be elucidated.

### Expression of the *phx1*^*+*^ gene increases at the stationary phase and by nutrient starvation

**Figure 3:**
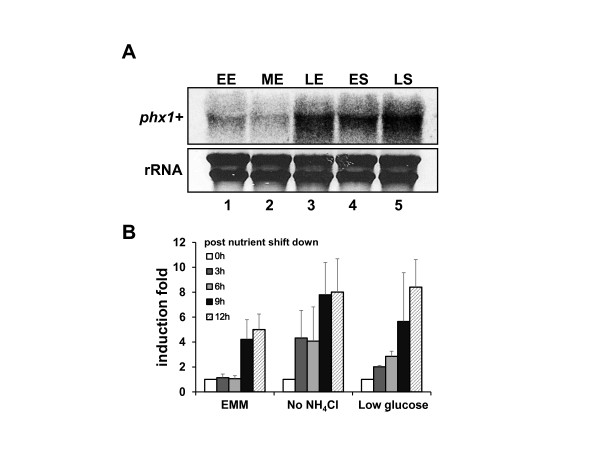
**Changes in *phx1* **^** *+* **^**mRNA level during vegetative cell growth and nutrient starved conditions.** (**A**) Expression profile of *phx1*^*+*^ gene during growth. RNA samples from wild type (JH43) cells grown in EMM for different lengths of culture time were analyzed for *phx1*^*+*^ mRNA by Northern blot. The sampling time corresponds to early exponential (EE, at around 12 h), mid-exponential (ME, 20 h), late exponential (LE, 28 h), early stationary (ES, 36 h), and late stationary (LS, 60 h) phases, following inoculation with freshly grown cells to an initial OD_600_ of 0.02. (**B**) Induction of *phx1*^*+*^ mRNA by nutrient starvation. Prototrophic wild type cells (972) were grown in EMM to OD_600_ of  0.5 ~ 1 and then transferred to modified EMM without NH_4_Cl (EMM-N) or with low (0.5%) glucose, for further incubation. At 3, 6, 9 and 12 h after media change, cells were taken for RNA analysis by qRT-PCR. The amount of *phx1*^*+*^ mRNA was measured by qRT-PCR, along with that of *act1*^*+*^ mRNA as an internal control. Average induction folds from three independent experiments were presented with standard deviations.

To monitor the expression profile of *phx1*^*+*^, we analyzed *phx1*^*+*^ transcripts at different growth phases by Northern blotting. As demonstrated in Figure 3A, the level of *phx1*^*+*^ transcripts was very low during early and mid-exponential phases (lanes 1 and 2). However, the level sharply increased during late exponential phase when cells approached the stationary phase (lane 3), and was maintained high during the stationary phase (lanes 4 and 5). This coincides with the fluorescence level from Phx1-GFP (Figure 1B), indicating that the level of Phx1 protein is determined largely by its transcript level.

Since cells enter the stationary phase when starved for nutrients [19,20], we examined the effect of nutrient shift-down during the exponential growth. For this purpose, prototrophic wild-type cells grown to mid-exponential phase in EMM were transferred to nitrogen-free EMM (EMM − N) or to low glucose EMM (EMM containing 0.5% glucose). The mRNA levels of *phx1*^*+*^ were measured by quantitative real-time PCR (qRT-PCR) along with the control *act1*^*+*^ mRNA. As demonstrated in Figure 3B, the relative level of *phx1*^*+*^ mRNA increased dramatically at earlier growth time in N-source or C-source limited conditions compared with the non-starved condition. These results indicate that the stationary-phase induction of *phx1*^*+*^ gene expression is due partly to nutrient starvation of N- or C-source.

### The *phx1*^*+*^ gene is required for long-term survival during the stationary phase and under nutrient-starved conditions

**Figure 4:**
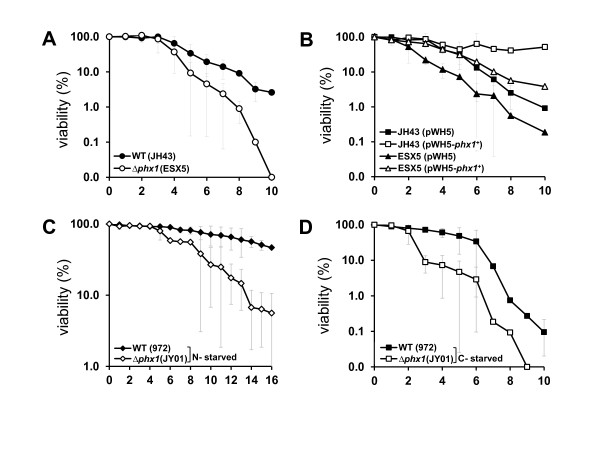
**Viability of *Δphx1* mutant cells in long-term culture.** Wild type and *Δphx1* mutant cells were grown in liquid EMM until they reached the stationary phase at OD_600_ of 8–9 (day 0). From this time point, aliquots were plated out on solid complex medium daily, and the surviving colonies were counted after 3 ~ 4 days of incubation at 30°C. At least three independent experiments were carried out to obtain survival curves for each strain. (**A**) The viability of wild type (JH43) and *Δphx1* mutant (ESX5) in EMM. (**B**) The viability in EMM of wild type (JH43) and *Δphx1* mutant cells containing pWH5 vector or pWH5-*phx1*^+^ plasmid. (**C, D**) The viability of prototrophic wild type (972) and *Δphx1*(JY01) in modified EMM without N-source (**C**) or with 0.5% glucose (**D**).

As *phx1*^*+*^ gene is induced during stationary phase and by nutrient starvation, we investigated its role in cell survival under those conditions. For this purpose, *Δphx1* null mutant was constructed and examined for its growth phenotype. The mutant strain did not show any significant difference in morphology, growth rate, or viability during the vegetative growth phase. However, when grown to the stationary phase in liquid EMM, *Δphx1* cells lost viability more quickly than the wild type when monitored by counting colony formation (Figure 4A). This defect in long-term viability of *Δphx1* mutant was rescued by ectopic expression of *phx1*^*+*^ (Figure 4B). In addition, overproduction of Phx1 in the wild-type strain greatly enhanced long-term viability (Figure 4B). Therefore, it is clear that Phx1 confers cells with fitness during long-term cultures, enhancing their survival rates. When the long-term survival experiments of Figure 4A were repeated with the strains (wild type 972 and *Δphx1* JY01) without auxotrophic markers, similar pattern was observed (data not shown).

We then examined the viability of *Δphx1* under nutrient-starved conditions. The wild type (strain 972) maintained its viability for a longer period of time in N-starved medium. In comparison, *Δphx1* (strain JY01) lost its viability at earlier time (Figure 4C). In C-starved condition as well, *Δphx1* lost its viability much quicker than the wild type (Figure 4D). Therefore, it appears clear that Phx1 serves a critical role in conferring fitness to the stationary-phase cells or cells under nutrient starvation, and thus enables them to maintain viability for longer period of time. Genetic studies have identified some genes that function in extending lifespan. In *S. pombe*, as in *S.cerevisiae*, cAMP/Pka1 and Sck2 signaling pathways have been shown to regulate chronological aging [21-23]. It has also been reported that respiration-defective mitochondrial dysfunction shortens chronological life span through elevating oxidative stresses [24,25]. Whether Phx1 is related with these signaling pathways and/or mitochondrial functions, and how, if it is, will be an interesting question to solve in the near future.

### Phx1 provides stress tolerance to oxidation and heat

**Figure 5:**
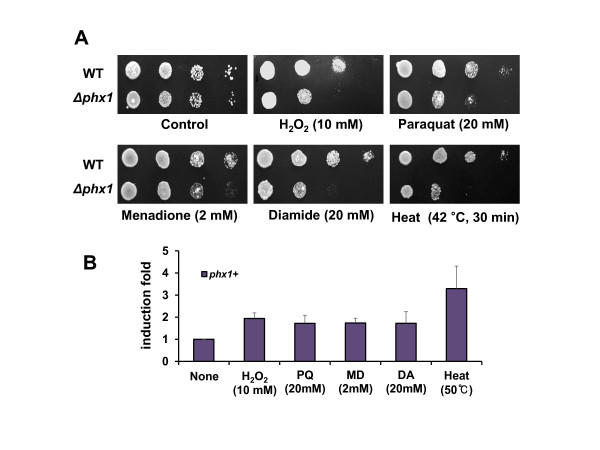
**Stress-sensitivity of *Δphx1* mutant and the inducibility of *phx1* **^** *+* **^**gene by various stresses.** (**A**) Stress-sensitivity of *Δphx1* mutant. To examine sensitivity of the wild-type (JH43) and *Δphx1* mutant to various oxidants and heat, exponentially growing cells in liquid EMM at 30°C were treated with 10 mM of H_2_O_2_, 20 mM of paraquat, 20 mM of diamide, or 2 mM menadione for 40 min each, or transferred to 42°C incubator for 30 min. Following stress treatment, equal number of cells were serially diluted, spotted onto EMM plates, and incubated at 30°C for 4 to 5 days. (**B**) Inducibility of *phx1*^*+*^ gene by various stresses. The wild-type (JH43) cells were grown to mid-exponential phase (OD_600_ of 0.5-1) in liquid EMM at 30°C, and treated with 10 mM hydrogen peroxide, 20 mM paraquat (PQ), 20 mM diamide (DA), or 2 mM menadione (MD) for 40 min each, or heat-shocked at 50°C for 30 min. RNA samples were analyzed for the level of *phx1*^*+*^ transcript in comparison with *act1*^*+*^, an internal control, by qRT-PCR. The average induction folds with standard deviations (error bars) from three independent experiments were presented.

It is widely accepted that cells in the stationary phase experience not only nutrient starvation, but also other stresses such as oxidation of cell components that include proteins and nucleic acids [26,27]. Therefore, stationary-phase cells activate various stress defense systems, and this defense is critical for long-term survival. To find out whether Phx1 is involved in tolerating various stresses other than nutrient starvation, we examined the sensitivity of *Δphx1* mutant to various oxidants and heat. As exemplified in Figure 5A *Δphx1* mutant became sensitive to oxidants such as H_2_O_2_ (peroxidation agent), paraquat and menadione (superoxide-generating agent), diamide (thiol-specific oxidant) and also to heat at 42°C. These results indicate clearly that Phx1 confers fitness to cells not only during nutrient starvation but also under oxidative and heat stress conditions. We analyzed whether these stress conditions induce the expression of the *phx1*^*+*^ gene by analyzing its RNA by qRT-PCR. The results in Figure 5B demonstrate that these acute stresses indeed elevated the level of *phx1*^+^ mRNA.

### The *Δphx1/Δphx1* diploid is defective in sporulation

**Figure 6:**
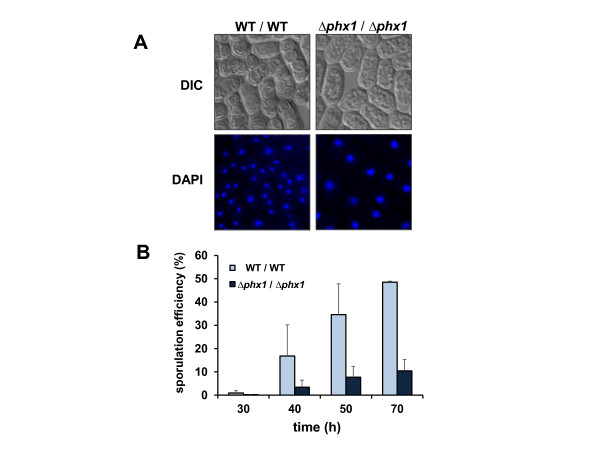
**Sporulation defect of *Δphx1/Δphx1* mutant diploid.** (**A**) The wild type and mutant diploid cells were grown to the stationary phase (OD_600_ of 8–9; ~70 h culture) in EMM at 30°C and examined under the microscope (Axiovert 200 M, Carl Zeiss). Representative DIC and DAPI images were presented. (**B**) Quantification of the sporulation efficiency. Diploid cells grown for different lengths of time at 30°C in EMM were examined under the microscope to count the number of spore-containing asci. The percentage of asci formation among a total of more than 500 counted cells was presented as sporulation efficiency. Cells grown from three independent cultures were examined to obtain average values.

When cells are starved of nutrients such as nitrogen or carbon sources, haploid yeast cells find other mating-type partners, conjugate to form diploids, which subsequently undergo meiotic division and sporulation. All of these sexual development processes are controlled by an extensive gene expression program [28,29]. A genome-wide analysis of *S. pombe* transcriptome has revealed that *phx1*^+^ (SPAC32A11.03 c) is one of the genes that are highly induced during meiotic spore formation [28]. This led us to examine whether Phx1 plays any role in meiosis. We first examined the mating efficiency of *Δphx1* mutant cells. Crossing *h*^*-*^ and *h*^*+*^ haploid *Δphx1* strains showed similar mating efficiency (54.2 ± 0.5%) to that of the wild type (56.7 ± 0.9%). Crossing between the wild type and *Δphx1* was similarly effective (53.1 ± 2.9%). This suggests that *Δphx1* mutation does not significantly impair conjugation and diploid formation. Therefore we obtained homozygous diploid strain *Δphx1/Δphx1* and examined the formation of tetrad meiotic spores by incubating in EMM. In comparison to the wild type, the mutant cells were very low in forming tetrad-containing asci, and mostly remained as cells with single large nucleus as monitored by DAPI staining (Figure 6A). Therefore, it appears that *Δphx1/Δphx1* diploid cells are defective in completing the first meiotic division [28]. The sporulation efficiency was determined by counting the number of asci among at least 500 cells counted. Compared with the wild-type cells which demonstrated up to about 50% sporulation efficiency, the mutant diploids exhibited only about 10% efficiency (Figure 6B).

## Conclusions

Phx1 is a homeobox-containing protein whose synthesis is elevated during the stationary phase. It resides primarily in the nucleus and contains the transcriptional activating ability when bound to DNA, supporting its role as a transcriptional regulator. Its synthesis is induced by nutrient starvation, various oxidative stresses, and by heat shock, coinciding with its role in long-term survival and stress resistance. It is also critically required for the formation of meiotic spores from diploid cells. Taken all these observations together, it is quite clear that Phx1 is a novel regulator that confers cells with fitness to survive during the nutrient-lacking stationary phase. It enhances viability and ability to form spores for the future, most likely through reprogramming gene expression pattern. Elucidation of the signaling pathway as well as its target genes will be of interest to understand the mechanism of long-term survival and sporulation specific in this fungi as well as common across other organisms.

## Methods

### Strains, plasmids and culture media

We used ED665 (*h*^*−*^*ade*6-*M*210 *leu132 ura4D18*), ED668 (*h*^+^*ade6M216 leu132 ura4D18*), JH43 (*h*^−^*ade6M210 leu132*) and 972 (*h*^*-*^) strains as the wild type [30]. To disrupt the *phx1*^*+*^ gene, we replaced 2200 nt of the *phx1*^*+*^ ORF in pUC18-*phx1*^*+*^ recombinant plasmid with a *ura4*^+^ cassette [31]. Digestion of pUC18*-Δphx1::ura4*^*+*^ with *Cla*I/*Bgl*II generated a 4.3 kb fragment, which was used to transform wild-type cells to create mutant strains ESX5 (*Δphx1::ura4*^*+*^ in ED665) and ESX8 (*Δphx1::ura4*^*+*^ in ED668). Transformants were confirmed by both Southern hybridization and PCR. We also generated the prototrophic *Δphx1* mutant without auxotrophic markers. For this purpose, ESX8 and an uracil auxotroph (*h*^*-*^*ura4D18*) [32] were mated, and a prototrophic *Δphx1* strain (JY01; *h*^*-*^*ura4D18 Δphx1::ura4*^*+*^) was obtained through spore selection and confirmation by PCR. For plasmids that express full-length Phx1, N-terminally truncated form (Phx1CD; 239–942 aa), and a hybrid form with Pap1 DNA-binding domain (Pap1DBD-Phx1CD; 1–117 aa of Pap1 linked with Phx1CD), appropriate DNA fragments were synthesized by PCR with specific primer pairs, using genomic DNA as a template and digested by proper restriction enzymes. For the hybrid form, the PCR fragments for Pap1DBD and Phx1CD were ligated. The final PCR products were cloned into multi-copy pREP42 vector [33]. pWH5-*phx1*^*+*^ was constructed by cloning the whole *phx1*^*+*^ gene with its own promoter into the *Hind*III-cut pWH5 plasmid [34]. All recombinant plasmids were confirmed by nucleotide sequencing. Growth and maintenance of *S. pombe* strains were generally done as described by Moreno *et al.*[35,36] in Edinburgh minimal medium (EMM) with appropriate supplements. Nitrogen-free medium was prepared by eliminating ammonium chloride (NH_4_Cl) from EMM whereas the low glucose medium contained only 0.5% of glucose, instead of 2% of glucose in EMM. For conjugation and sporulation, malt extract (ME) medium (3% malt extract) was used.

### Construction and intracellular localization of Phx1-GFP fusion protein

A C-terminal 1535 nt of the *phx1*^*+*^ gene (*ΔNTphx1*) was generated by PCR, digested with *Nde*I and *BamH*I, and cloned in front of the EGFP gene in pRIP42*EGFP-C*[37] to allow GFP-fusion at the C-terminus. For chromosomal integration, the recombinant plasmid was linearized by *Kp*nI at a site within the *phx1*^*+*^ gene and transformed into ED665 strain. The correct integrant (ESXF5; *phx1*^+^*EGFP/ΔNTphx1::ura4*^+^ in ED665) created by double crossing-over was selected through *ura4*^*+*^ marker and confirmed by both Southern hybridization and PCR. The fusion strain was grown in EMM to exponential or stationary phase, and was examined for GFP signal. The fluorescence and DIC (differential interference contrast) images of the living cells were captured by Zeiss Axiovert 200 M microscope. Representative images from more than three separate experiments were presented. 

### Northern blot analysis

RNA samples prepared from EMM-grown cells at different conditions were separated on agarose gels containing formaldehyde, and transferred onto a Hybond-N^+^ membrane (Amersham) for hybridization. Gene-specific probes for *phx1*^*+*^, *ctt1*^*+*^*, trr1*^*+*^*,* and *gpx1*^*+*^ genes were generated by PCR and radio-actively labeled as recommended by the manufacturer. After hybridization, signals were visualized and quantified by PhosphorImager (BAS-5000) with Multi Gauge (Fuji) program.

### Quantitative real-time PCR

Each RNA sample (1 μg/μl) was reverse-transcribed into cDNA using RevertAid™ Reverse Transcriptase kit (Fermentas). PCR was performed with SYBR Green/ROX qPCR master mix (Fermentas) using gene-specific primers for *phx1*^*+*^ and *act1*^*+*^ which serves as an internal control. Triplicate PCRs with gene-specific primer pairs for each gene were carried out as recommended by the manufacturer, using a quantitative real-time PCR machine (ABI PRISM®Sequence Detection System, Applied Biosystems) with analysis software SDS2.2 (Applied Biosystems).

### Cell survival assay

To measure chronological life span, cells were inoculated at initial OD_600_ of 0.02 in liquid EMM, and grown until OD_600_ reached the maximum value of about 8 to 9. From this time point (day 0), aliquots were taken daily and plated on complex (YES for auxotrophs and YE for prototrophs) solid medium, following appropriate dilutions to plate out similar number of cells. Cell colonies were counted after 3 to 4 days incubation at 30°C. The viable cell count at day 0 was regarded as 100% survival rate. For nutrient-specific starvation, cells grown to OD_600_ of 0.5 to 1 in liquid EMM were washed with sterile distilled water, and resuspended in EMM without NH_4_Cl or EMM with 0.5% instead of 2% glucose. Following 24-hr further incubation at 30°C, cells were grown on solid YE medium to count colonies as described above.

### Stress sensitivity

For oxidative stress, hydrogen peroxide (Fluka), superoxide generators paraquat (methyl viologen; sigma) and menadione (vitamin K3, non-salt form from ICN), and a thiol-specific oxidant diamide (sigma) were used. Heat was treated at 42°C (for cell viability) or 50°C (for transcriptional induction). All the acute stresses were applied to exponentially grown cells in liquid EMM (OD_600_ 0.5-1) for 40 or 30 min (heat shock). The stress-treated cells were spotted on EMM solid media for sensitivity analysis, or harvested for RNA preparation to examine *phx1*^*+*^ induction.

### Sporulation assay

Pairs of ED665 (*h*^*-*^) and ED668 (*h*^*+*^), as well as ESX5 (*Δphx1*, *h*^*-*^) and ESX8 (*Δphx1*, *h*^*+*^), were mated with each other on ME plate and incubated at 25°C for 2 days. Diploid cells were selected for the complementing markers on EMM. Following growth to the stationary phase in liquid EMM, the formation of asci that contain tetrad spores was examined by microscopy, following nuclear staining by DAPI. Three independent experiments were carried out to quantify the efficiency of ascus formation. At least 500 cells in each culture were counted.

## Authors’ contributions

JYK designed and performed the majority of the experiments. ESK designed and performed some experiments. All the authors contributed to analyzing and interpreting results. JYK and JHR wrote, read, and approved the final manuscript.
